# Detection of insecticide resistance and knockdown resistance (*kdr*) mutations in *Aedes albopictus* across different regions

**DOI:** 10.3389/fcimb.2026.1892686

**Published:** 2026-08-12

**Authors:** Nuo Xu, Xiao Pan, Kun Peng, Hao Yin, Yingchun Yang, Chenxin Han, Maoqing Gong, Hongmei Liu

**Affiliations:** 1 Digestive Disease Hospital of Shandong First Medical University, Shandong Institute of Parasitic Diseases, Jining, China; 2 Shandong First Medical University & Shandong Academy of Medical Sciences, Jinan, China

**Keywords:** *Aedes albopictus*, insecticide resistance, *kdr*, mosquito control, *VGSC*

## Abstract

**Objective:**

This study aimed to monitor the resistance status of adult *Aedes albopictus* (*Ae. albopictus*) to commonly used insecticides in Jining (Shandong Province), Guangzhou (Guangdong Province), and Haikou (Hainan Province), and to provide evidence for improving vector control strategies.

**Methods:**

Between May and July 2024–2025, field-collected *Ae. albopictus* larvae from the three regions were reared under laboratory conditions. Adult insecticide susceptibility was assessed using the WHO tube test. Species identity was confirmed morphologically under a dissecting microscope. Genomic DNA was extracted from individual female mosquitoes, and fragments of the voltage-gated sodium channel (*VGSC*) gene were amplified by PCR and sequenced. *kdr* mutations were analyzed using BioEdit software.

**Results:**

Across the three regions, 24-hour mortality rates of *Ae. albopictus* exposed to 0.03% deltamethrin, 0.4% permethrin, and 0.08% lambda-cyhalothrin were all below 80%, indicating resistance. For 0.05% propoxur, mortality rates were 100%, 79.21%, and 98.17% in Jining, Guangzhou, and Haikou, respectively. For 0.5% malathion, mortality rates were 88.12%, 98.06%, and 88.12%, respectively. In Guangzhou, mortality rates for 0.08% beta-cyfluthrin and cis-cypermethrin were both below 80%, while in Jining and Haikou, they ranged from 86.17% to 93.88%. Mutations were detected at *VGSC* codons 1016, 1532, and 1534 in all three populations. The GGA/G allele at codon 1016 was found only in Guangzhou (4.17%). At codon 1532, a single mutant allele (ACC/T) was detected (2.43%), with three genotypes observed: I/I (96.12%), I/T (2.91%), and T/T (0.97%). At codon 1534, five alleles were identified: TTC (F) (35.44%), TCC (S) (24.27%), TGC (C) (38.35%), TTG (L) (1.46%), and CTC (L) (0.49%), forming seven genotypes, including F/F (18.44%), F/C (6.80%), F/S (23.30%), F/L (3.88%), S/S (7.77%), C/C (30.10%), and S/C (9.71%).

**Conclusion:**

The studied *Ae. albopictus* populations showed high resistance to three commonly used pyrethroid insecticides, while relatively lower resistance was observed for propoxur and malathion. *kdr* mutation frequencies varied across regions, with generally low mutation rates at codon 1016. The mutation frequency at codon 1534 was lower in Jining than in Guangzhou and Haikou, whereas codon 1532 showed a relatively higher mutation frequency in Jining. These findings highlight the importance of rational insecticide use, continuous resistance monitoring, and rotation of insecticides to delay resistance development and spread.

## 1 Introduction

*Aedes albopictus* (Skuse, 1894) (Diptera: Culicidae) is a mosquito species capable of transmitting several mosquito-borne diseases, including dengue fever and chikungunya (Xie et al., 2025). With global warming and accelerated urbanization, the geographical distribution of *Ae. albopictus* has expanded significantly worldwide, gradually spreading from tropical and subtropical regions to higher-latitude temperate areas (Yao et al., 2020). The species has been detected not only in warm southern regions such as Hainan and Guangdong Province, but also in colder regions including the Tibet Autonomous Region and Liaoning Province. Similarly, dengue outbreaks have been reported in Shandong Province in 2017 and 2019, further highlighting the public health threat posed by *Ae. albopictus.* Owing to its wide distribution and strong ecological adaptability, this species has become a major challenge for global public health (Lambrechts et al., 2010; Huang et al., 2023; Jiao et al., 2025). Dengue fever remains one of the most significant mosquito-borne diseases, with an estimated 390 million infections annually and approximately 40 million symptomatic cases worldwide (Shepard et al., 2016; Harapan et al., 2020; Kularatne and Dalugama, 2022). Regarding the history of dengue in the study areas: In Guangzhou, a major city in the study area, over 3,000 cases were reported in 2024 (Dai et al., 2025). In Hainan Province, 363 cases were reported in 2023, with 17 of those cases occurring in Haikou City (Gu et al., 2026). For Shandong Province, the most recent available official data shows 63 cases in 2019 and 104 cases in 2017 (Public Health Science Data Center, https://www.phsciencedata.cn/Share/edtShareNew.jsp?id=39311).

Chemical insecticides play a central role in mosquito-borne disease control. However, inappropriate and prolonged use of insecticides has contributed to the emergence and spread of resistance in mosquito populations (Rose, 2001; Zhang et al., 2024b). Previous studies have shown that *Ae. albopictus* in China has developed high levels of resistance to pyrethroid insecticides, whereas resistance to carbamate and organophosphate insecticides remains relatively low (Zhao et al., 2022a). Insecticide resistance in *Ae. albopictus* has developed gradually through long-term exposure and natural selection. The underlying mechanisms mainly include metabolic resistance, cuticular resistance, target-site resistance, and behavioral resistance (South and Hastings, 2018; Asgarian et al., 2023). Pyrethroids primarily target the voltage-gated sodium channel (*VGSC*) gene, and mutations in this gene can lead to *kdr*, reducing mosquito susceptibility to pyrethroids (Zang et al., 2023). Research on *kdr* mutations has focused mainly on codons 1016 in domain II and 1532 and 1534 in domain III of the *VGSC* gene. Mutations at these sites are widely distributed, and combined mutations at multiple loci can significantly reduce *VGSC* sensitivity to pyrethroids (Smith et al., 2016). Notably, the F1534S and F1534C mutations are commonly reported variants in Chinese *Ae. albopictus* populations. Although the I1532T mutation has been detected in multiple regions across China (such as Beijing, Shanghai, and Guizhou), its frequency is generally low (for example, only 0.53% in Guizhou) (Zhao et al., 2022b; Zhang et al., 2024a). Furthermore, the combined mutations of V1016G or I1532T with F1534S are associated with higher levels of pyrethroid resistance (Liu et al., 2025).

In this study, *Ae. albopictus* samples were collected from three regions representing different temperature zones to assess their resistance to pyrethroid, carbamate, and organophosphate insecticides. The frequency of *kdr* mutations was investigated across these regions. The findings are intended to support evidence-based guidance for insecticide rotation strategies and contribute to the development of more effective vector control and mosquito-borne disease prevention measures.

## 2 Materials and methods

### 2.1 Insecticide resistance assessment

#### 2.1.1 Mosquito collection and laboratory rearing

Larvae of *Ae. albopictus* were collected between May and July 2024–2025 from Jining (Shandong Province), Guangzhou (Guangdong Province), and Haikou (Hainan Province), representing warm temperate, subtropical, and tropical climatic regions, respectively. Approximately 500 larvae were collected from the wild in each geographic population, and there were no fewer than 10 breeding sites at each sampling location.

Download the annual average temperature and annual precipitation data for each sampling point from 2010 to 2025 using the WheatA software (**Supplementary Table S1**).

#### 2.1.2 Insecticides and reagents used for bioassays

Larvae were reared to the F1 generation, and adult female mosquitoes that had emerged for 3–5 days without blood-feeding were selected for adult bioassays. Larvae were fed a mixture of pig liver powder and yeast powder at a ratio of 1:3, and adult mosquitoes were provided with 10% glucose solution. Detailed records of sampling sites and collection information were maintained for all field locations (**Table 1**). Following collection, larvae were transported to the laboratory and reared under standardized insectary conditions at 26 ± 1 °C, 70 ± 5% relative humidity, and a 12-hour light/dark photoperiod.

**Table 1:** Details of *Ae. albopictus* sampling sites collected from May to July 2024–2025.

Sampling location	Sampling period	Latitude and longitude	Environment type	Habitat type
Jining, Shandong (SDJN)	May–July 2024–2025	35.409456°N, 116.587533°E	Residential area	Discarded plastic buckets and vases
Guangzhou, Guangdong (GDGZ)		23.09672°N, 113.29840°E	School	Disused water storage containers and flower beds
Haikou, Hainan (HNHK)		19.94242°N, 110.46075°E	Urban area	Waste tyres, polystyrene boxes

The insecticides used for susceptibility testing included 0.03% deltamethrin, 0.4% permethrin, 0.08% lambda-cyhalothrin, 0.08% beta-cyfluthrin, 0.08% cis-cypermethrin, 0.05% propoxur, and 0.5% malathion. Insecticide-impregnated papers were obtained from the Vector Control Laboratory of the Institute of Infectious Disease Prevention and Control, Chinese Center for Disease Control and Prevention. Analytical-grade acetone was supplied by the Fine Chemicals Plant in Laiyang Economic and Technological Development Zone.

#### 2.1.3 WHO tube bioassay for insecticide susceptibility testing

The experimental procedures strictly followed the World Health Organization (WHO)-recommended tube contact bioassay (WHO, 2016). Briefly, 25–30 non-blood-fed female mosquitoes (3–5 days old) were placed in exposure tubes lined with insecticide-impregnated filter paper and exposed for 60 minutes. Following exposure, the mosquitoes were transferred to holding tubes and provided with 10% glucose solution. Mortality was recorded 24-hour post-exposure, and the mortality rate was calculated for each group. Each experiment was repeated 3–4 times, a mixture of white oil and diethyl ether was used as the untreated control group. Resistance status was classified according to the diagnostic-dose criteria specified in GB/T 26347-2010: Populations demonstrating mortality rates of 98–100% were classified as susceptible, 80–97% as possibly resistant, and below 80% as resistant. No correction is necessary when the control mortality is below 5%. Abbott’s formula is applied when the control mortality falls between 5% and 20%. In cases where the control mortality exceeds 20%, the experiment is deemed invalid and the bioassay should be repeated.

### 2.2 *kdr* genotyping

#### 2.2.1 Identification of adult mosquitoes

Adult *Ae. albopictus* specimens collected from three field sites were examined under a dissecting microscope, morphologically confirmed as *Ae. albopictus*, preserved in 95% ethanol, and stored at −80 °C for downstream analysis.

#### 2.2.2 Genomic DNA extraction from individual mosquitoes

Female *Ae. albopictus* specimens that survived the bioassay were randomly selected from three sampling sites for genetic analysis. Genomic DNA was extracted from individual female mosquitoes using the Cador^®^ Pathogen 96 QIAcube^®^ HT system with a commercial kit (Cat. No. 51331, QIAGEN). Briefly, Individual specimens were placed into 1.5 mL Eppendorf tubes containing lysis beads and subjected to two grinding steps. A total of 200 µL lysis buffer and 20 µL proteinase K were added, followed by vortex mixing. Samples were incubated at 56 °C for 2–3 h and centrifuged, and the supernatant was transferred to a sample plate. The purified DNA was stored at –80 °C for downstream analysis.

#### 2.2.3 PCR amplification and sequencing of *VGSC kdr* regions

Primers were designed to amplify partial sequences of the second and third transmembrane domains of the *VGSC* gene based on previous studies (Kasai et al., 2011, 2019).

The Domain III region containing codons I1532 and F1534 was amplified using primers aegSCF7 (5’-GAGAACTCGCCGATGAACTT-3’) and aegSCR7 (5’-GACGACGAAATCCAGGT-3’) in a 20 µL PCR system containing 10 µL 2× Phanta Max Master Mix, 1 µL DNA template, 1 µL forward primer, 1 µL reverse primer, and 7 µL RNase-free water. The cycling conditions included initial denaturation at 95 °C for 3 min, followed by 32 cycles of denaturation at 95 °C for 15 s, annealing at 55 °C for 15 s, and extension at 72 °C for 1 min, with a final extension at 72 °C for 5 min. After amplification, products were stored at 4 °C.

The Domain II fragment containing codon S989, I1011, L1014 and 1016 was amplified using primers aegSCF20 (5’-GACAAT GTG GAT GGC TCC CC-3’) and aegSCR21 (5’-GCA ATC TGG CTT GTT AAC TTG-3’) in a 25 μL reaction mixture containing 12.5 μL 2× Phanta Max Master Mix, 2 μL DNA template, 1 μL of each primer, and 8.5 μL RNase-free water. PCR conditions included initial denaturation at 95 °C for 3 min, followed by 30 cycles of 95 °C for 30 s, 58 °C for 30 s, and 72 °C for 45 s, with a final extension at 72 °C for 5 min. PCR products were verified by agarose gel electrophoresis, and positive amplicons were subjected to Sanger sequencing at a commercial sequencing facility.

#### 2.2.4 Sequence alignment and mutation analysis

The obtained sequences were compared with the National Center for Biotechnology Information (NCBI) database using BLAST, with results showing more than 99% identity to *Ae. albopictus*, confirming species-level identity. The obtained sequences were aligned with the *Ae. albopictus kdr* gene reference sequence (GenBank accession No. AY663384.1) using BioEdit software. Following alignment, mutations at each locus were recorded and analyzed. Pearson correlation analysis was performed using SPSS 26 to examine the relationships between the insecticide resistance phenotypes of adult *Ae. albopictus* and the mutation frequencies at the resistance-associated loci in three regions.

## 3 Results

### 3.1 Insecticide resistance profiles of *Ae. albopictus*

**Figure 1:**
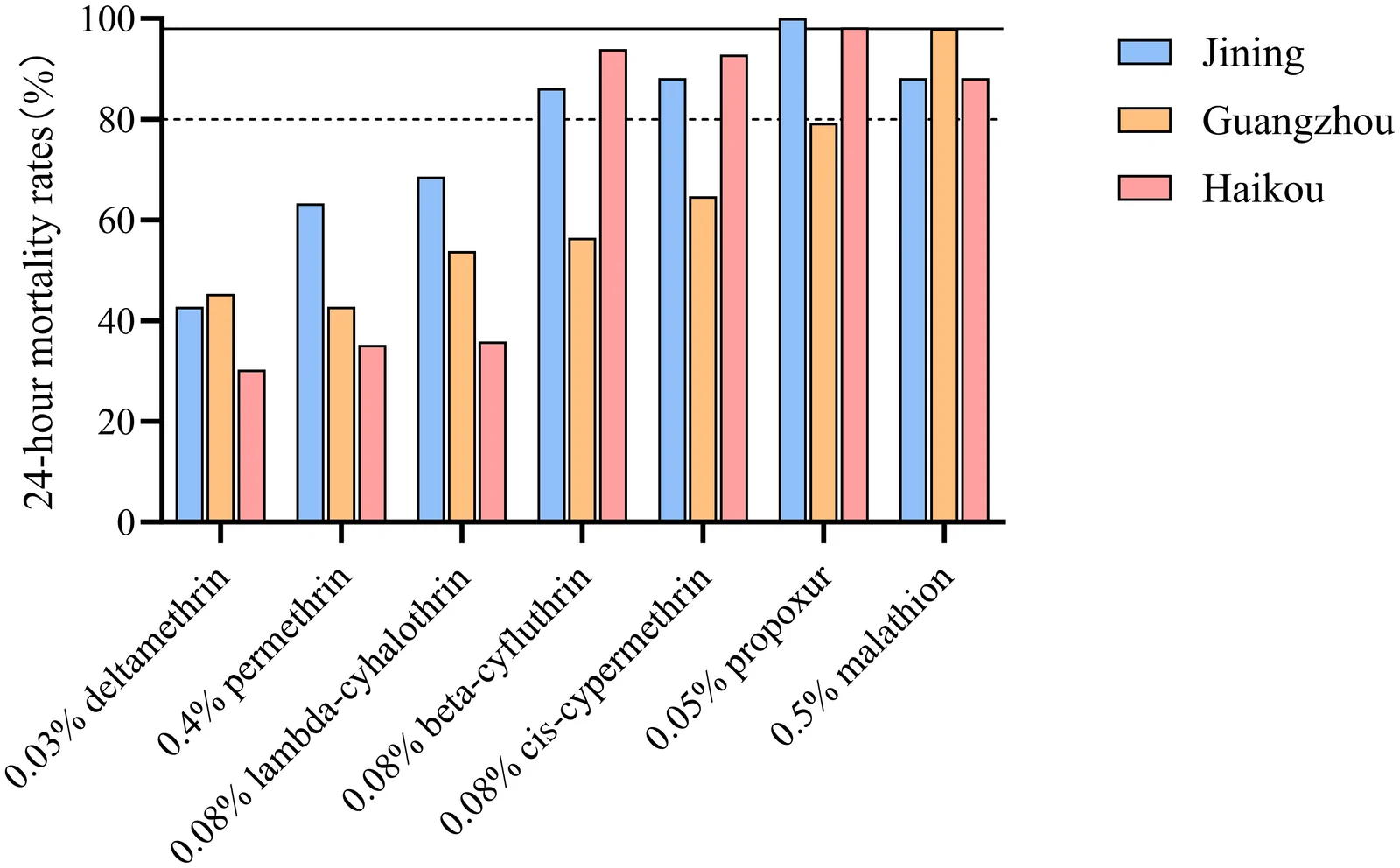
Resistance levels of three geographic strains of *Ae. albopictus* to seven insecticides. Dashed line: 80% mortality, solid line: 98% mortality.

A control group was included in each experiment, no mortality was observed in any of the control groups, so Abbott’s correction was not required. The resistance status of *Ae. albopictus* populations from the three study regions against seven insecticides are summarized in **Table 2**. In Jining, Guangzhou, and Haikou, 24-hour mortality rates following exposure to 0.03% deltamethrin, 0.4% permethrin, and 0.08% lambda-cyhalothrin were all below 80%, indicating resistance in all three populations. For 0.05% propoxur, mortality rates were 100% in Jining, 79.21% in Guangzhou, and 98.17% in Haikou, indicating resistance in Guangzhou. For 0.5% malathion, mortality rates were 88.12% in Jining, 98.06% in Guangzhou, and 88.12% in Haikou, indicating susceptibility in Guangzhou. For 0.08% beta-cyfluthrin and cis-cypermethrin, Guangzhou exhibited mortality rates of 56.48% and 64.65%, respectively, indicating resistance. Guangzhou showed resistance to multiple insecticides, with susceptibility observed only for 0.5% malathion (**Figure 1**).

**Table 2:** Insecticide resistance status of adult *Ae. albopictus* in three study regions.

Location	Insecticide	Test mosquitoes (n)	Knockdowns (only)	1-hour knockdown rate (%)	Deaths (number)	24-hour mortality rate (%)	Resistance status
Jining	0.03% deltamethrin	96	40	41.67	41	42.41	Resistance
	0.4% permethrin	98	46	46.94	62	63.27	Resistance
	0.08% lambda-cyhalothrin	86	62	72.09	59	68.60	Resistance
	0.08%beta-cyfluthrin	94	77	81.91	81	86.17	Potential resistance
	0.08% cis-cypermethrin	101	71	70.30	89	88.12	Potential resistance
	0.05% propoxur	96	96	100.00	96	100.00	Sensitive
	0.5% malathion	101	71	70.30	89	88.12	Potential resistance
Guangzhou	0.03% deltamethrin	95	39	41.05	43	45.26	Resistance
	0.4% permethrin	103	37	35.92	44	42.72	Resistance
	0.08% lambda-cyhalothrin	93	33	35.48	50	53.76	Resistance
	0.08% beta-cyfluthrin	108	43	39.81	61	56.48	Resistance
	0.08% cis-cypermethrin	99	48	48.48	64	64.65	Resistance
	0.05% propoxur	101	94	93.07	80	79.21	Resistance
	0.5% malathion	103	91	88.35	101	98.06	Sensitive
Haikou	0.03%deltamethrin	96	25	26.04	29	30.21	Resistance
	0.4% permethrin	108	33	30.56	38	35.19	Resistance
	0.08% lambda-cyhalothrin	106	44	41.51	38	35.85	Resistance
	0.08% beta-cyfluthrin	98	69	70.41	92	93.88	Potential resistance
	0.08% cis-cypermethrin	83	69	83.13	77	92.77	Potential resistance
	0.05% propoxur	109	109	100.00	107	98.17	Sensitive
	0.5% malathion	101	87	86.14	88.12	88.12	Potential resistance

### 3.2 Alleles and genotype frequencies of *VGSC kdr* mutations

#### 3.2.1 V1016 mutation frequency in *VGSC*

Among the three *Ae. albopictus* populations, no mutations were detected at the S989, I1011, and L1014 sites. The GGA(G) mutant allele at codon 1016 was detected only in the Guangzhou population, with a frequency of 4.17%. The predominant genotype was wild-type homozygote V/V (97.62%), followed by heterozygote V/G (2.38%), while no mutant homozygote G/G was detected (**Table 3**).

**Table 3:** Genotype and allele frequencies at the *VGSC* codon 1016 site in three *Ae. albopictus* populations.

Location	Allele frequencies		Genotype frequencies		
	GTA/V (%)	GGA/G (%)	V/V (%)	G/G (%)	V/G (%)
Jining	28 (100)	0 (0)	14 (100)	0 (0)	0 (0)
Guangzhou	23 (95.83)	1 (4.17)	11 (91.67)	0 (0)	1 (8.33)
Haikou	32 (100)	0 (0)	16 (100)	0 (0)	0 (0)
Total	83 (98.81)	1 (1.19)	41 (97.62)	0 (0)	1 (2.38)

#### 3.2.2 *VGSC* mutations at codons 1532 and 1534

**Figure 2:**
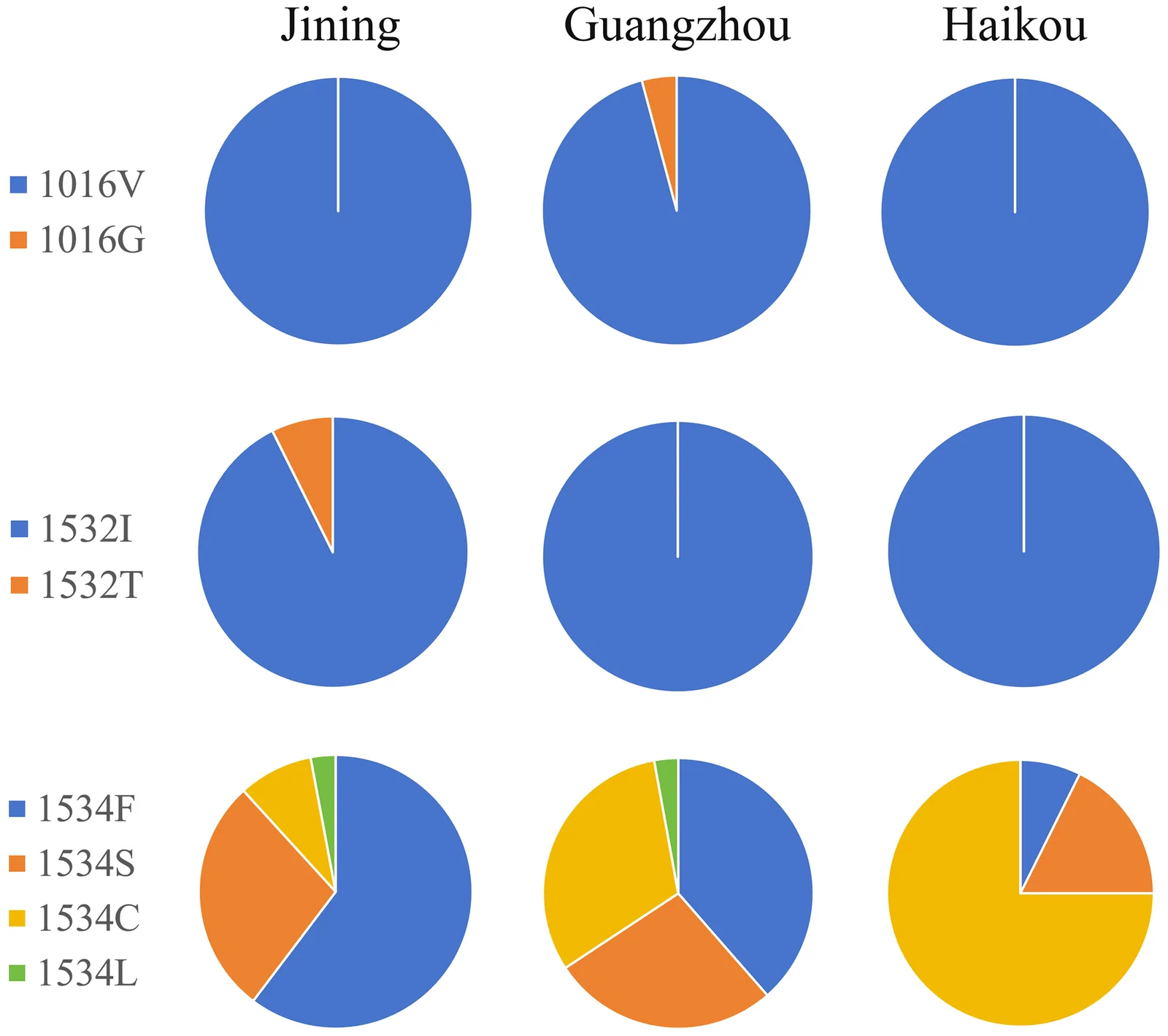
Allele frequency distributions at loci (1016, 1532, and 1534) of the *VGSC* gene in the three geographical strains.

A total of 103 *Ae. albopictus* individuals were analyzed for mutations in the *VGSC* domain III, encompassing codons 1532 and 1534. At codon 1532, two alleles were identified: ATC (I) with a frequency of 97.57% and ACC (T) with a frequency of 2.43%. At codon 1534, five alleles were detected: TTC (F) (35.44%), TCC (S) (24.27%), TGC (C) (38.35%), TTG (L) (1.46%), and CTC (L) (0.49%) (**Table 4**; **Figure 2**).

**Table 4:** Allele frequencies at codon positions 1532 and 1534 of the *VGSC* gene in three *Ae. albopictus* populations.

Location	Samples (n)	I1532		F1534				
		ATC/I (%)	ACC/T (%)	TTC/F (%)	TCC/S (%)	TGC/C (%)	TTG/L (%)	CTC/L (%)
Jining	68	63 (92.65)	5 (7.35)	41 (60.29)	19 (27.94)	6 (8.82)	1 (1.47)	1 (1.47)
Guangzhou	70	70 (100)	0 (0)	27 (38.57)	19 (27.14)	22 (31.43)	2 (2.86)	0 (0)
Haikou	68	68 (100)	0 (0)	5 (7.35)	12 (17.65)	51 (75.00)	0 (0)	0 (0)
Total	206	201 (97.57)	5 (2.43)	73 (35.44)	50 (24.27)	79 (38.35)	3 (1.46)	1 (0.49)

Gene distribution at codon 1532 consisted of wild-type homozygote I/I (96.12%), heterozygote I/T (2.91%), and mutant homozygote T/T (0.97%). At codon 1534, seven genotypes were observed: wild-type F/F (18.44%); heterozygotes F/C (6.80%), F/S (23.30%), and F/L (3.88%); homozygous variants S/S (7.77%) and C/C (30.10%); and heterozygote S/C (9.71%). Overall, 81.56% of *Ae. albopictus* individuals carried at least one variant allele at codon 1534 of the *VGSC* gene (**Table 5**). In this study, six genotypes were identified at codon 1534 in both the Jining and Guangzhou regions. In Jining, the F/S genotype was predominant, whereas in Guangzhou, the C/C genotype was most frequent. In Haikou, only four genotypes were observed, with the C/C genotype being the most common. All populations exhibited varying levels of resistance to pyrethroid insecticides.

**Table 5:** Genotype frequencies at codon positions 1532 and 1534 of the *VGSC* Gene in three *Ae. albopictus* populations.

Location	Samples (n)	I1532			F1534						
		I/I (%)	T/T (%)	I/T (%)	F/F (%)	S/S (%)	C/C (%)	F/C (%)	F/S (%)	F/L (%)	C/S (%)
Jining	34	30 (88.24)	1 (2.94)	3 (8.82)	12 (35.29)	3 (8.82)	2 (5.88)	2 (5.88)	13 (38.24)	2 (5.88)	0 (0)
Guangzhou	35	35 (100.00)	0 (0)	0 (0)	7 (20.00)	5 (14.29)	10 (28.57)	2 (5.71)	9 (25.71)	2 (5.71)	0 (0)
Haikou	34	34 (100)	0 (0)	0 (0)	0 (0)	0 (0)	19 (55.88)	3 (8.82)	2 (5.88)	0 (0)	10 (29.41)
Total	103	99 (96.12)	1 (0.97)	3 (2.91)	19 (18.44)	8 (7.77)	31 (30.10%)	7 (6.80)	24 (23.30)	4 (3.88)	10 (9.71)

### 3.3 Correlation analysis between resistance phenotypes and mutation frequencies of resistance-associated genes in *Ae. albopictus*

Pearson correlation analysis revealed that (**Supplementary Table S2**) no significant correlations were observed between the mutation frequencies of each mutant allele at the *kdr* 1534 locus (1534S, 1534C, 1534L, and the total mutations 1534S/C/L) and the resistance phenotypes of adult *Ae. albopictus* to the five pyrethroid insecticides across the three regions (*P* > 0.05). However, significant positive correlations were detected between the 24-hour mortality rates of adult *Ae. albopictus* exposed to 0.08% beta-cyfluthrin and those exposed to 0.08% cis-cypermethrin across the different regions (*r* = 0.999, *P* < 0.05), as well as between the mutation frequencies of 1534S and 1534L (*r* = 0.999, *P* < 0.05). These findings suggest that interactions among different insecticides may exist and might synergistically contribute to the development of resistance in *Ae. albopictus*; moreover, the mutant alleles at the *kdr* 1534 locus appear to be interrelated.

## 4 Discussion

*Ae. albopictus* is one of the primary vectors of dengue fever, and insecticide-based control remains a key strategy for limiting the spread of mosquito-borne diseases. However, the extensive and prolonged use of insecticides has driven the emergence of resistance in mosquito populations, making insecticide resistance a major challenge in vector control programs (Dalpadado et al., 2021). Therefore, assessing insecticide resistance and *kdr* mutations in *Ae. albopictus* across different regions is essential for improving vector control strategies and reducing disease transmission risk (Wan-Norafikah et al., 2023). In this study, insecticide susceptibility in three regions was evaluated alongside *kdr* mutation frequencies in wild populations of *Ae. albopictus*, providing relevant data for resistance management.

Pyrethroid insecticides are widely used in mosquito control, particularly during outbreaks of mosquito-borne diseases, which has contributed to the increasing prevalence of resistance in mosquito populations (Karunaratne et al., 2013; Hamainza et al., 2016; Li et al., 2021). Previous studies have reported high levels of pyrethroid resistance in *Ae. albopictus* across multiple regions of China (Chen et al., 2016; Li et al., 2018; Yang et al., 2020; Zhao et al., 2022a). In this study, *Ae. albopictus* from all three regions showed resistance to 0.03% deltamethrin, 0.4% permethrin, and 0.08% lambda-cyhalothrin, while showing relatively higher susceptibility to organophosphate and carbamate insecticides. These findings are consistent with previous reports from different geographic locations (Yang et al., 2020; Li et al., 2021; Wu et al., 2021; Deng et al., 2024; Wu et al., 2024). Variation in resistance levels among the three regions may be associated with differences in insecticide usage patterns, application intensity, and local dengue transmission intensity. The Jining population exhibited comparatively higher susceptibility to insecticides than populations from Guangzhou and Haikou.

In recent years, target-site resistance mechanisms have received increasing attention, with *kdr* mutations in the *VGSC* gene recognized as a major contributor to pyrethroid resistance (Zang et al., 2023; Qi et al., 2024). This study demonstrated substantial variation in *kdr* mutation patterns across populations and genomic loci (1016, 1532, and 1534). Among the three study regions, the mutation frequency at codon 1532 was higher in Jining and lower in Guangzhou and Haikou regions, consistent with previous reports (Gao et al., 2018; Wei et al., 2021; Zhao et al., 2022b). Codon 1534 exhibited high genetic diversity, with multiple mutant alleles and genotypes detected, resulting in an overall mutation frequency of 81.56%. The presence of multiple allelic forms at this locus indicates increasing genetic diversification of *kdr*-associated mutations in Chinese *Ae. albopictus* populations and suggest a polymorphic genetic structure (Wei et al., 2021). Previous studies have reported an association between the F1534S mutation and pyrethroid resistance phenotypes (Chen et al., 2016). The F1534C mutation has been shown to be associated with higher levels of resistance to Type I and Type II pyrethroid insecticides, and exhibits particularly strong resistance to commonly used compounds such as deltamethrin (Gao et al., 2018; Kasai et al., 2019). The F1534C mutation predominates in Guangzhou and Haikou. In 2014, a dengue fever outbreak occurred in Guangzhou. The F1534C mutation was predominantly detected in Guangzhou and Haikou. In 2014, a dengue fever outbreak occurred in Guangzhou. To control the vector *Ae. albopictus* during this outbreak, over 27,000 kg of pyrethroid insecticides were applied via ultra-low volume (ULV) spraying alone, along with substantial quantities of temephos and fenthion as larvicides (Yiguan et al., 2017; Li et al., 2018). Results from a resident questionnaire survey conducted at five research sites in Hainan Province show that pyrethroids are the most widely used for mosquito control, organophosphate insecticides are primarily used for agricultural pest control, and carbamate are used for mosquito control only by a small number of households at the Haikou site (Li et al., 2021). Dengue fever outbreaks occurred in Shandong Province in 2017 and 2019. In the Jining region, the long-term, widespread, and diverse use of insecticides may have led to the accumulation of multiple resistance alleles under intense selective pressure; therefore, this region should be prioritized for resistance monitoring and control, and diverse pest management strategies should be implemented to slow the further development of resistance (Yue et al., 2019; Liu et al., 2020). The pesticides used vary by region, and the resulting differential selective pressures may have shaped the regional distribution patterns of the *kdr* mutation.

Environmental factors may also influence *kdr* mutation dynamics. Climatic factors such as temperature and precipitation directly affect mosquito growth and reproduction, while also influencing the residual levels and degradation rates of pesticides in the environment. For example, warm and humid climatic conditions promote mosquito growth and development, which may lead to higher mutation rates and increased selection for pesticide resistance (Chen et al., 2021). reported a positive correlation between the F1534 mutation frequency and annual average temperature (AAT), while the I1532 mutation showed a negative correlation with AAT. Between 2010 and 2025, the AAT in Jining, Guangzhou, and Haikou were 15.3 °C, 23.5 °C, and 24.6 °C, respectively, while the average annual precipitation was 752.2 mm, 2,191.4 mm, and 1,505.6 mm, respectively. This study observed higher 1534 mutation frequencies in Guangzhou and Haikou compared with Jining, while the opposite trend was observed for codon 1532. Similarly, populations in southern China with higher AAT exhibited higher levels of insecticide resistance than those in northern regions (Zhao et al., 2022b). further reported that mutations at positions 1016, 1532, and 1534 are associated with climatic factors and dengue endemicity, with a negative correlation between AAT and mutation frequency at codon 1016. In this study, mutation frequencies at codon 1016 remained low across all regions.

Continuous insecticide application is likely contributing to increasing resistance levels in *Ae. albopictus* populations. The presence of multiple *kdr* mutations, particularly at codon 1534, highlights the growing complexity of resistance mechanisms. Given that Guangzhou and Haikou are dengue-endemic regions and Jining has also reported outbreaks in recent years, ongoing monitoring of insecticide resistance and *kdr* mutation frequencies is essential for guiding evidence-based vector control strategies and preventing future dengue outbreaks. Several limitations of this study should be acknowledged. First, the mosquitoes used for *kdr* genotyping were selected from individuals that survived the adult bioassay at each sampling site, whereas dead individuals were not analyzed. Second, although our data revealed geographical variation in both resistance phenotypes and mutation frequencies, the lack of a statistically significant correlation between genotype and phenotype may be attributable to the limited number of sampling sites. In addition, our investigation was confined to the *kdr* resistance mechanism, while metabolic resistance and cuticular resistance (both of which are critical factors in pyrethroid resistance) were not examined. Therefore, further studies with larger sample sizes and broader coverage of resistance mechanisms are warranted.

## Data Availability

The raw data supporting the conclusions of this article will be made available by the authors, without undue reservation.
