# Supplementary material for: Detection of insecticide resistance and knockdown resistance (kdr) mutations in Aedes albopictus across different regions
Source: Front Cell Infect Microbiol. 2026 Aug 12;16:1892686. doi: 10.3389/fcimb.2026.1892686 (PMC13506523; doi:10.3389/fcimb.2026.1892686)
Supplement: Supplementary file 1 [file Table1.docx]

**Table S1.** The annual average temperature (AAT) of collection sites

| **Population** | **Province** | **City** | **Weather station** | **Station number** | **Valid period** | **AAT**  **(℃)** | **Annual precipitation**  **（mm）** |
| --- | --- | --- | --- | --- | --- | --- | --- |
| SDJN | Shandong | Jining | Yanzhou | 54916099999 | 2010~2025 | 15.3 | 752.2 |
| GDGZ | Guangdong | Guangzhou | Baiyun international | 59287099999 | 2010~2025 | 23.5 | 2191.4 |
| HNHK | Hainan | Haikou | Haikou | 59758099999 | 2010~2025 | 24.6 | 1505.6 |

Note: Abbreviations: AAT, annual average temperature;

**Table S2.** Analysis of the Correlation Between Mutation Frequencies at the *kdr* 1534 Locus in *Ae. albopictus* and Resistance to Pyrethroid Insecticides

|  |  | Insecticide | | | | | | | Mutant | | | |
| --- | --- | --- | --- | --- | --- | --- | --- | --- | --- | --- | --- | --- |
| Item |  | 0.03% deltamethrin | 0.4% permethrin | 0.08% lambda-cyhalothrin | 0.08%  beta-cyfluthrin | 0.08% cis-cypermethrin | 0.05% propoxur | 0.5% malathion | 1534S | 1534C | 1534L | 1534S/C/L |
| Insecticide | 0.03% deltamethrin | 1 |  |  |  |  |  |  |  |  |  |  |
|  | 0.4% permethrin | 0.586 | 1 |  |  |  |  |  |  |  |  |  |
|  | 0.08% lambda-cyhalothrin | 0.809 | 0.951 | 1 |  |  |  |  |  |  |  |  |
|  | 0.08% beta-cyfluthrin | -0.77 | 0.065 | -0.248 | 1 |  |  |  |  |  |  |  |
|  | 0.08% cis-cypermethrin | -0.743 | 0.107 | -0.207 | 0.999* | 1 |  |  |  |  |  |  |
|  | 0.05% propoxur | -0.567 | 0.335 | 0.026 | 0.962 | 0.973 | 1 |  |  |  |  |  |
|  | 0.5% malathion | 0.631 | -0.259 | 0.054 | -0.981 | -0.988 | -0.997 | 1 |  |  |  |  |
| Mutant | 1534S | 0.974 | 0.755 | 0.921 | -0.605 | -0.572 | -0.365 | 0.438 | 1 |  |  |  |
|  | 1534C | -0.877 | -0.904 | -0.992 | 0.368 | 0.329 | 0.101 | -0.18 | -0.963 | 1 |  |  |
|  | 1534L | 0.983 | 0.724 | 0.902 | -0.641 | -0.609 | -0.408 | 0.479 | 0.999* | -0.95 | 1 |  |
|  | 1534S/C/L | 0.981 | 0.416 | 0.678 | -0.88 | -0.86 | -0.718 | 0.771 | 0.911 | -0.765 | 0.929 | 1 |

Note: The * in the Pearson correlation signifies a noteworthy correlation at the 0.05 level (two-sided).
